# Ni tolerance and its distinguished amelioration by chelating agents is reflected in root radius of *B. napus* cultivars

**DOI:** 10.1007/s00709-018-1287-0

**Published:** 2018-07-25

**Authors:** Humera Nawaz, Stephan Manhalter, Aamir Ali, Muhammad Yasin Ashraf, Ingeborg Lang

**Affiliations:** 1 Cell Imaging and Ultrastructure Research, University of Vienna, Althanstrasse 14, 1090 Vienna, Austria; 2 Department of Botany, University of Sargodha, Sargodha, 40100 Pakistan; 3 Nuclear Institute for Agriculture and Biology, P.O Box 28, Jhang Road, Faisalabad, Pakistan

**Keywords:** *Brassica napus*, Canola cultivars, Chelating agent, Citric acid, EDTA, Metal tolerance

## Abstract

The negative effect of excess nickel (Ni) on plants is well investigated but there is only little information on its influence on root anatomy and a possible amelioration by chelating agents. In this study, we utilized light microscopy to observe anatomical changes in canola (*Brassica napus*) roots and investigated the element content by X-ray microanalysis. Ni-tolerant (Con-II) and Ni-sensitive cultivars (Oscar) were selected for this purpose. The plants were treated with 30 ppm NiSO_4_. Then, citric acid and ethylene-diamine-tetra-acetic acid (EDTA) (alone or in combination) were applied to observe the influence of chelating agents in metal stress amelioration. Ni treatment led to significant swelling of the roots in the Con-II variety as compared to the cultivar Oscar. Application of EDTA reduced the root radius of Con-II plants and this effect for Ni tolerance is discussed. According to X-ray microanalyses, Ni ions were more dispersed in the sensitive cultivar as indicated by metal adsorption to the cell wall. We investigate the hypothesis that an enhanced capacity of binding metals to the cell wall allows the plants to tolerate more heavy metals.

## Introduction

Heavy metal pollution is one of the most common ecological problems on a global scale (Doumett et al. 2008). Many toxic effects of heavy metals have been reported on plants and animals as well as humans (Järup 2003). Among the different metal pollutants, nickel (Ni) has gained considerable attention in recent years because of its rapidly increasing concentration in soil, air, and water (Ahmad and Ashraf 2011). Ni is deposited in the environment by factory waste, field irrigation with high heavy metal content, transfer of heavy metals from mine tailing, and steady application of organic and mineral fertilizers, pesticides, and other anthropogenic sources (Orlov et al. 2002).

In low concentration, Ni fulfills a variety of essential roles in plants (Eskew et al. 1984) and plants cannot complete their life cycle without it. Ni is also a constituent of important enzymes, such as urease. Therefore, Ni deficiency leads to several developmental defects in plants, e.g., reduced growth, senescence, chlorosis, alteration in nitrogen metabolism, or reduced iron uptake (Sirko and Brodzik 2000). At elevated concentrations, on the other hand, Ni is highly phytotoxic leading also to negative effects on growth, photosynthesis, and membrane function (Madhava Rao and Sresty 2000).

Phytoremediation is a new technique in which hyperaccumulator plant species are used to remove heavy metals from the soil (Kirkham 2006; Park et al. 2012). It is a low-cost and eco-friendly technology based on plants (Mueller et al. 1999). Canola (*Brassica napus*) is a well-documented phyto-accumulator and phyto-extractor (Croes et al. 2015) and was successfully used for phytoremediation of different heavy metals due to its great general metal uptake capacity, fast growth, and high biomass production (Vamerali et al. 2010; Kumar et al. 1995). Apart from hyperaccumulating properties, *B. napus* is an important crop plant. It is cultivated worldwide as a source of vegetable oil for biodiesel. Furthermore, it grows well on rather poor soils (Rashid and Anwar 2008; Szczygłowska et al. 2011).

A plant’s uptake of heavy metals can be enhanced by the application of chelating agents (Huang et al. 1997). These substances improve metal uptake by increasing the mobility and solubility of a certain metal in the soil thereby also boosting metal absorption. In return, chelators can significantly minimize the level of free metal ions. Chelating agents can be synthetic, like diethylene-triamine-penta-acetic-acid (DTPA) and ethylene-diamine-tetra-acetic-acid (EDTA), or organic, like citric acid (CA) (Sinha et al. 2013). Organic chelating agents are biodegradable and pose a lower leaching hazard as compared to their synthetic counterparts (Bareen 2012).

Here, we investigated the effects of two chelating agents, EDTA and CA, in ameliorating Ni stress in two *B. napus* cultivars, a tolerant variety (Con-II) and a sensitive one (Oscar). Following the hypothesis that metal stress causes a premature development of endodermis and vascular tissue in the root tips, we tested fresh roots for their central cylinder–cortex ratios. The presumed, accompanying adsorption of Ni at the root tips was semi-quantitatively measured by X-ray microanalysis at the scanning electron microscopy level.

## Material and methods

### Plant material

Seeds of *B. napus* were obtained from Ayub Agriculture Research Institute, Faisalabad, Pakistan. Two varieties, Con-II and Oscar, were selected due to their tolerance in salt stress (Ulfat et al. 2007) and yield loss/oil content experiments (Sana et al. 2003). Preliminary studies for Ni tolerance showed that Con-II was more tolerant than Oscar (H.N. unpublished data). Subsequently, in the present study, the two varieties are referred to “tolerant” (Con-II) and “sensitive” (Oscar) and further studies are underway to confirm this tolerance. The seeds were surface sterilized with 5% HCl solution and washed several times with tap water, followed by distilled water. The sterilized seeds were sown in pots containing rough quartz sand (Min2c, 0.5–2.0 mm). Ni treatment was applied at the time of sowing as NiSO_4_ (30 ppm). EDTA (1.5 mM) and CA (10 mM) applications, separately and in combination, were done after 2 weeks of Ni treatment. After 30 days of culture, the seedlings were harvested. Three independent replicates of the experiment were performed.

### Fresh root staining

To compare the radius of the whole root and the central cylinder in treated and non-treated samples, fresh root tips of six individuals per treatment (about 2 cm long) were collected and placed into patent blue dye (0.5%) for 20 min to label the vascular tissue. After rinsing in water, the root tips were placed on glass slides, mounted in water, and covered with a cover slip. The diameter of the whole root and the central cylinder were measured at a distance of 150 and 300 μm from the tip under a light microscope and then divided by two for radius values. Between 14 and 16 stained fresh roots per treatment were analyzed in the light microscope.

### Light microscopy

The fresh root tips were photographed at a microscope stand (Nikon Eclipse Ni) with a digital camera (Nikon DS-Ri2) using the objectives × 4, × 10, × 20, and × 40 as well as NIS-Elements BR software (Nikon).

### Scanning electron microscope and energy dispersive X-ray spectroscopy

To investigate the Ni adsorption as well as fluctuations in Ni caused by the different treatments along the root tip, energy dispersive X-ray spectroscopy (EDX) was employed. Fresh root tips (1 cm) were affixed with sticky carbon foil on aluminum stubs and air dried at room temperature for 24 h. The dry samples were carbon-coated (Leica Med 020). The root tips were then analyzed in a scanning electron microscope (SEM) (Jeol IT 300) and semi-quantitative measurements of element contents were taken as line scans along the sample, starting from the tip and traveling over a distance of at least 600 μm in 5-μm steps. The magnification was set to × 160. The detector and supported TEAM software were both from the EDAX company.

### Quantification of Ni content

The total amount of Ni in roots was measured by inductively coupled plasma mass spectroscopy (ICP-OES; Optima 2100DV, Perkin–Elmer) at the Pakistan Institute of Nuclear Science and Technology, Islamabad, Pakistan. The plants were grown and treated as described above and harvested after 30 days. Fresh roots were separated from the plant and dried in an oven at 45 °C for 48 h. Dry roots were ground to powder. 0.1 g of ground root material was transferred into digesting tubes and digested according to the method of Wolf (1982).

### Solution state modeling

Solution state equilibria of NiSO_4_ treatments as well as EDTA, CA, and the combinations thereof were modeled with Visual MINTEQ V3.1 (MINTEQ) (Gustaffson 2018; Sassmann et al. 2015). Equilibrium state concentrations of all chemical species in the solution were calculated using the included default MINTEQ databases. The pH and ionic strengths were automatically calculated.

### Statistics and software

Statistical analyses were performed using Statgraphics XVI software (StatPoint Technologies Inc. 2010). Since the values were not always normally distributed, the Mann–Whitney *U* test was used to determine significant differences between the samples. For general purposes, Microsoft Office was employed and Adobe Creative Suite 4 was used to edit the images and create image plates.

## Results

### Root radius and differentiated tissue thickness

**Fig. 1:**
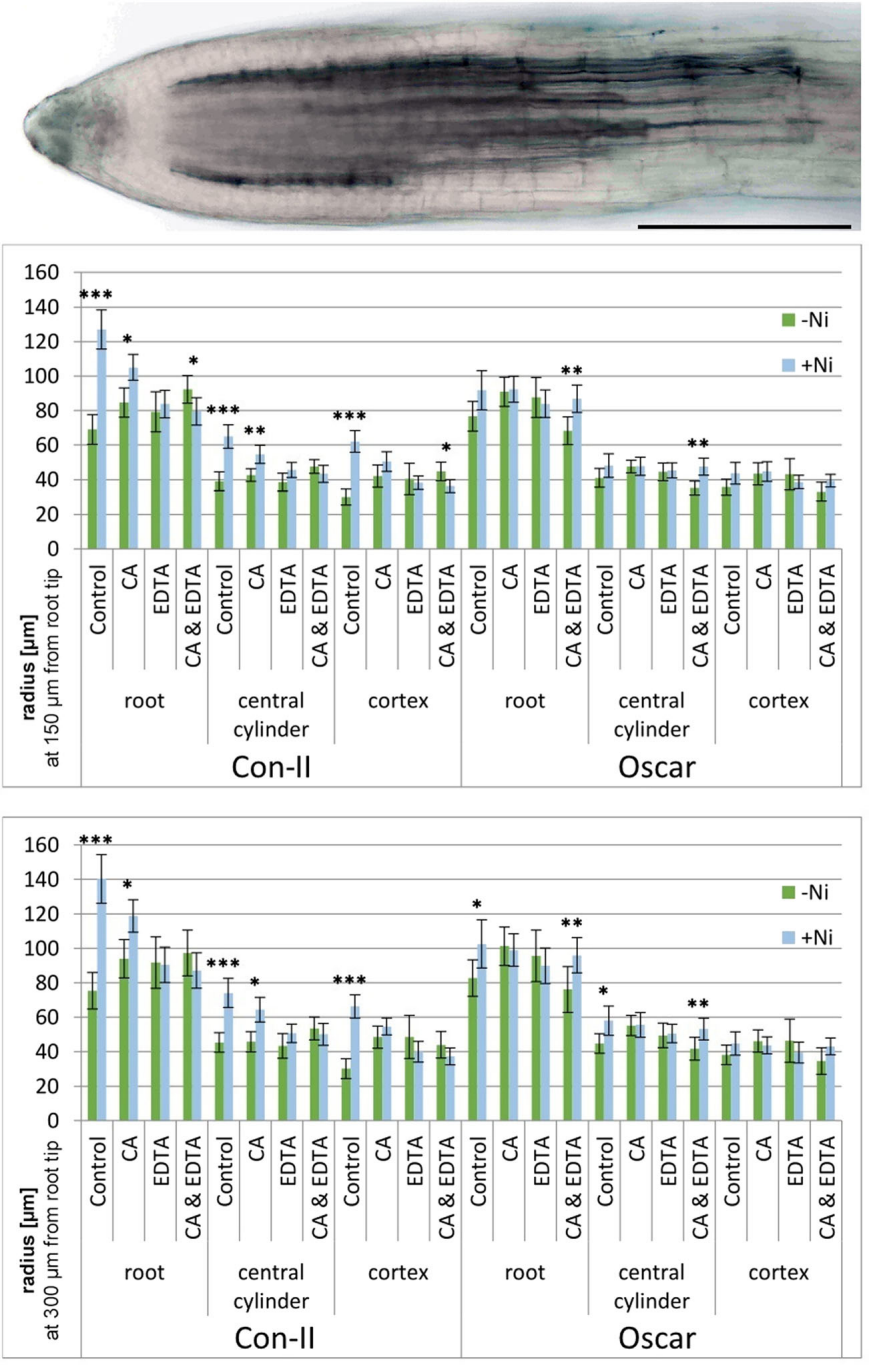
Analyses of fresh root tips of 4-week-old *B. napus* seedlings treated with Ni, citric acid (CA), and EDTA. **a** Light-microscopic image of *B. napus* root tip, cultivar Oscar treated with CA and stained with patent blue. Bar: 150 μm. **b** Radius of whole roots, central cylinder, and root cortex measured at 150-μm distance from the tip. **c** Radius of whole roots, central cylinder, and root cortex measured at 300-μm distance from the tip. 95% confidence interval, *n* = 14–16. Asterisks denote significant difference between medians *p < 0.05, **p < 0.01, ***p < 0.001

In fresh roots of the sensitive *B. napus* variety, Oscar, and the tolerant one, Con-II, we tested the thickness of the cortex and central cylinder after Ni treatment and investigated the role of chelating agents for root anatomy. Fresh roots were stained in patent blue for the identification of vascular tissue (Fig. 1a) and compared to the non-labeled cortex. Furthermore, we determined the development of vascular tissue by its thickness at certain distances from the root tip. At 150 and 300 μm from the tip, the radius of the whole root of Con-II plants increased significantly with Ni treatment compared to the control (*p* < 0.001, Fig. 1b, c). Root radius was also slightly increased in the combined Ni and CA treatment of the Con-II cultivar at both distances when compared to the samples only treated with CA (*p* < 0.05). In the Con-II samples treated with Ni and CA + EDTA, the root radius was slightly lower than without Ni, particularly at 150 μm from the tip (*p* < 0.05). The radius of the central cylinder in Con-II increased significantly in the Ni treatment, compared to the control, both at 150- and 300-μm distance from the root tip (*p* < 0.001, Fig. 1b, c). Similarly, the cortex increased in the combined Ni and CA treatment when compared to CA only. The effect was slightly more significant at 150 μm from the tip (*p* < 0.01; Fig. 1b), than at 300 μm (*p* < 0.05; Fig. 1c). The cortex of Con-II is highly significantly thicker in the Ni-treated samples, compared to the control, at both 150- and 300-μm distance from the tip (*p* < 0.001 both, Fig. 1b, c). At 150 μm, the thickness of the cortex of the Ni-, CA-, and EDTA-treated samples decreased compared to the samples treated with CA and EDTA (*p* < 0.05). Overall, there is a trend of radius decrease from Ni only (control) > CA > EDTA > CA + EDTA in the Ni-treated samples.

In the sensitive variety Oscar, the radius of the roots showed an increase in the Ni treatment, compared to the control, but it was only significant at 300-μm distance from the tip (*p* < 0.05, Fig. 1c) and the increase was less that in the tolerant variety. The combined treatment of Oscar with CA + EDTA caused a significant increase in root radius after Ni treatment at both measured distances (*p* < 0.01 both). This pattern is different to Con-II, the tolerant variety. The radius of the central cylinder of Oscar was increased in the Ni-treated samples, compared to the control, but it was only significant at 300-μm distance from the tip (*p* < 0.05, Fig. 1c). In the Ni samples treated with CA + EDTA, we found a significant increase in the radius of the central cylinder, compared to samples treated with CA + EDTA only. This was true for both measured distances from the root tip (*p* < 0.01). No significant differences in cortex thickness were found in this cultivar.

In summary, Ni treatment caused significantly thicker roots in the tolerant variety Con-II as compared to the more sensitive cultivar Oscar. EDTA application (alone or in combination with CA) reduced the radius of Con-II plants treated with Ni.

### Ni localization along root tips

**Fig. 2:**
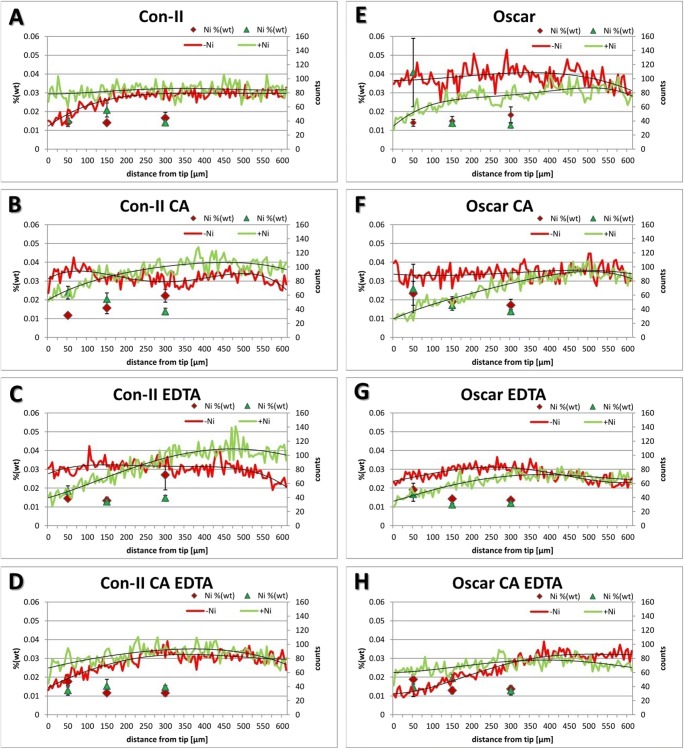
Semiquantiative analyses of Ni in root tips of Con-II and Oscar cultivars after treatment with Ni as well as CA, EDTA, and CA–EDTA. The green and red lines illustrate the actual, detected X-ray quanta (counts) with a 4-degree polynomic trend line along the measured line starting at the root tip (*n* = 2). The diamond (◊) and triangle (Δ) show local averages of Ni contents at 50, 150, and 300 μm from the root tip in weight % (*n* = 10). **a**–**d** Con-II seedlings (tolerant variety); **e**–**h** Oscar seedlings (sensitive cultivar)

To determine the actual adsorption of Ni along the root tips, we used energy dispersive X-ray spectroscopy (EDX) at the scanning electron microscopy (SEM) level. This method allows for the semi-quantitative detection of Ni on dry tissue. Fresh root tips were placed on aluminum stubs on sticky carbon foil and allowed to dry at room temperature. After carbon coating, line scans were performed in two biological duplicates of each treatment. The scans started at the root tip and stretched up to 600 μm. At certain distance points, i.e., 50, 150, and 300 μm from the root tip of each respective treatment (◊, Δ), the data were used to calculate the semi-quantitative Ni content (wt%; Fig. 2). The red and green curves in Fig. 2 illustrate the actual amount of X-ray quanta with the specific energy to identify Ni in the sample. The black lines represent the respective trend curves. In general, the Ni content was very low and close to the detection limit of SEM–EDX. However, there was a distinctive pattern in the measured X-ray quanta. Ni-treated Con-II samples (tolerant variety) had a stable count of Ni-specific X-rays along the whole length of 600 μm. The Con-II samples without Ni treatment started with lower counts but plateaued almost at the same height as the Ni-treated samples, i.e., around 150–200 μm from the root tip (Fig. 2a). The Con-II samples that were treated with Ni and only one chelating agent (CA, Fig. 2b or EDTA, Fig. 2c) started at a lower level and peaked slightly above the control values. In these samples, the Ni-free roots produced a rather flat trend line which dropped down slightly at both ends (Fig. 2b, c). The curves of Ni and non-Ni treatments cross each other at about 150 μm from the tip for CA treatments (Fig. 2b) and at 235 μm from the tip for EDTA treatments (Fig. 2c). Line scans of Con-II samples treated with CA + EDTA were similar to samples without any chelating agents (Fig. 2a); the lines become close at 300 μm and converge towards 600 μm from the root tip (Fig. 2d).

In the sensitive cultivar Oscar, the wt% of Ni was high in the very tip (50 μm) of the Ni-treated samples (◊) but the line scans of Ni treatments start at a lower level than the control samples without Ni. The lines converged at 550–600 μm (Fig. 2e). The treatments with only one chelating agent showed a similar crossing pattern as Con-II but the lines came close or crossed further away from the tip, at ~ 480 μm for CA (Fig. 2f) and ~ 430 μm for EDTA (Fig. 2g**)**, respectively. Oscar root tips treated with CA + EDTA had a slightly higher starting count for Ni but at about 325 μm from the tip, it dropped below the line of the Ni-free samples (Fig. 2h).

### Total Ni content in roots

**Fig. 3:**
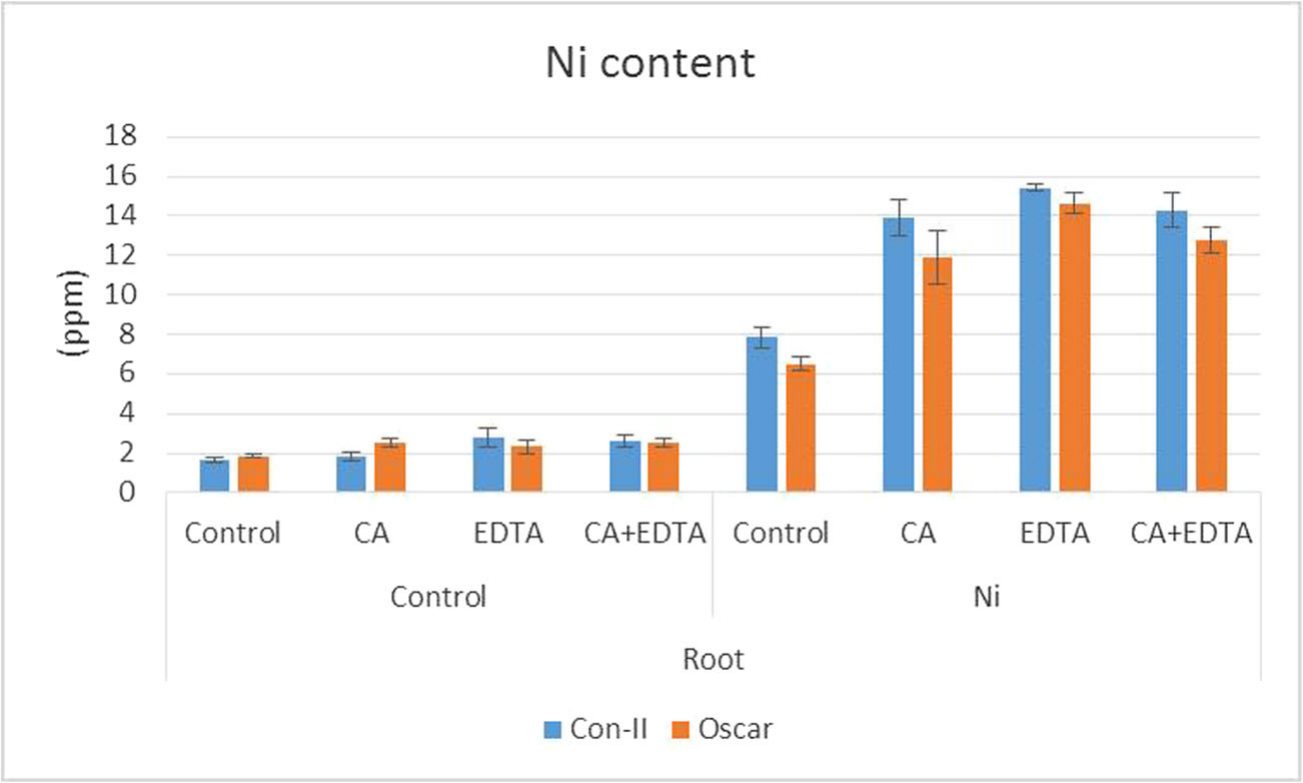
ICP-OES values of total Ni (ppm) in roots of the cultivars Con-II and Oscar. In control (without Ni), the Ni content is similar in all treatments (control, CA, EDTA, and CA + EDTA, respectively) and significantly lower than after Ni application (Ni). In Ni-treated samples, chelating agents enhanced Ni content in roots and Ni content in Oscar is always lower than in Con-II

As expected and verified by ICP-OES measurements, Ni treatment caused highly significantly greater amounts of Ni (from 6.5 to 15.4 ppm) in roots of both varieties than in controls without Ni application (*p* < 0.001). The results are graphically depicted in Fig. 3. Without Ni treatment, the Ni content was similar in all plants and always below 2.8 ppm (control, CA, EDTA, and CA + EDTA, respectively). By contrast, Ni application in combination with CA, EDTA, and CA + EDTA caused an increased content of Ni when compared to Ni-treated controls without chelating agents (*p* < 0.001).

The Ni content also differed between the cultivars Con-II and Oscar in the Ni-treated samples. Ni content in Con-II is always higher than in Oscar (*p* < 0.01 for Ni only, CA and CA + EDTA, and *p* < 0.05 for EDTA treatments). The higher amount of Ni in Con-II is pointing towards greater adsorption or accumulation capacities than in Oscar.

### Metal availability

In fertilizers, metals are often linked to EDTA and CA for better availability. These chelators, however, could also cause a reduction of free metal ions, thereby minimizing the effect of Ni. We therefore estimated the availability of free Ni ions with the modeling program MINTEQ for equilibrium state concentrations (Table 1). Our data confirmed an almost full dissociation of the ionic salt NiSO_4_ into Ni^2+^ (99.855%) and SO_4_^−2^ (99.989%). Only a very small portion below 0.1% remained as NiSO_4_ (aq) or NiOH^+^, respectively (Table 1). Both chelators, CA and EDTA, very effectively bound free Ni ions. CA bound 12.3% of Ni as Ni–citrate^−^ and 87.7% as Ni–(citrate)_2_^−4^. EDTA complexed 97.3% as NiEDTA^−^ and 2.7% as Ni–OH–EDTA^−3^. Also, in the combined treatment of EDTA + CA, Ni was predominantly associated with EDTA (96.9% NiEDTA^−^ and 3.1% Ni–OH–EDTA^−3^) and not with CA. In none of the chelator treatments, the simulation software detected free Ni ions (Table 1). However, the *B. napus* seeds were treated with NiSO_4_ at the time of sowing, 2 weeks before the chelators were added. During this time, NiSO_4_ is fully dissociated and the metal available is Ni^2+^.

**Table 1:** Equilibrium state concentrations of all chemical species in the external solution of NiSO_4_, as well as its combinations with citric acid (CA) and EDTA treatments, calculated by Visual MINTEQ

Component	Species	% of the total concentration in treatment			
		NiSO4	NiSO4 + CA	NiSO4 + EDTA	NiSO4 + CA + EDTA
SO_4_^−2^	SO_4_^−2^	99.989	100.000	100.000	100.000
	NiSO4 (aq)	0.010			
Ni^+2^	Ni^+2^	99.855			
	NiSO4 (aq)	0.018			
	NiOH^+^	0.126			
	NiH–(citrate)2^−3^		0.034		
	Ni–citrate^−^		12.267		
	Ni–(citrate)_2−_^4^		87.696		
	NiEDTA^−2^			97.351	96.871
	NiOHEDTA^−3^			2.649	3.129
Citrate^−3^	Citrate^−3^		99.848		0.033
	H–citrate^−2^		0.142		99.998
EDTA^−4^	EDTA^−4^			57.058	65.876
	HEDTA(ii)^−3^			42.907	34.090
	NiEDTA^−2^			0.033	0.033

## Discussion

The root of a plant is the first organ to encounter heavy metals in the soil and thus, roots have been widely studied to assess the impact of stress factors. Accumulation of Ni in roots is greatly enhanced by Ni application (ICP-OES measurements; Fig. 3) and Ni treatment caused morphoanatomical changes when compared with the control samples. This led us to believe that there was a negative impact of the administered Ni on the roots, as reported by Maruthi Sridhar et al. (2007). A thorough anatomical study of cross sections would require precautious handling of the delicate roots during fixation, staining, embedding, and sectioning processes that are likely to cause structural changes and preparation artifacts. Therefore, we focus on the interpretation of the data gained in fresh roots (Fig. 1).

Our investigations of fresh roots showed that Ni treatment increased root radius, central cylinder radius, and cortex thickness in Con-II, the tolerant *B. napus* variety. Gomes et al. (2011) and Maksimović et al. (2007) mentioned an increase in thickness of certain root tissues as a strategy to minimize the translocation of metals. According to Fitter (2002), this can be caused by suberization and lignification of the cortex cells thereby reducing the uptake of water and nutrients. In addition, the cytoskeleton is involved in a wide variety of cellular functions such as cell division, cell expansion, cell wall synthesis, organelle movement, and tip growth (Seagull 1989). Heavy metals like Al disrupt the normal functioning of cytoskeleton which results in growth inhibition and substantial swelling of the root apex (Baskin et al. 1994). Moreover, an inhibition of root growth is accompanied by an increase in root diameter suggesting that the plant cytoskeleton may also be a target of heavy metal toxicity due to change in water balance and nutrient absorption under heavy metal stress (Zobel et al. 2007). Here, we observed an increase in root diameter by Ni treatment in the tolerant *B. napus* variety. Therefore, the development of thick roots could be a strategy to minimize the translocation of metals, because thicker roots provide a greater area for retention of heavy metals and decrease their translocation to other parts of the plant. In rice, the high tolerance to heavy metals is characterized by high proportions of exodermis and endodermis tissue in the roots (Lux et al. 2004). The use of chelating agents in ameliorating the effect of Ni showed that the application of EDTA was more effective than CA in Con-II; the combination of chelating agents caused no synergistic effects.

In the sensitive variety Oscar, Ni treatment caused a lower increase of root radius indicating a correlation of root thickness with Ni sensitivity. Interestingly, in this variety, CA and EDTA used individually did not affect the root radius. The application of EDTA and CA has the capacity to increase the metal accumulation but more accumulation was noted by application of EDTA (Sinhal et al. 2010). The combined application of CA + EDTA caused most increase in root radius in Ni-treated samples. Chelating agents could create a barrier for heavy metals and enhance the plasticity of root anatomy. On the other hand, EDTA and CA can destabilize cell membranes resulting in a general loss and increased permeability. This enhanced membrane permeability to solutes can cause oxidative damage (Filek et al. 2012; Nakazawa and Nagatsuka 1980) as well as changes in the structure and orientation of membrane lipids (Catala 2012). Free EDTA has a higher damaging effect than EDTA chelates due to their binding with membrane stabilizing cations (Vassil et al. 1998). This is in contrast to the findings presented here where chelating agents appear to have an effect on root thickness, particularly in the tolerant *B. napus* variety, which we rather link to amelioration than damage.

Changes in cell shape and organization as well as the early development of vascular tissue and endodermis suggest that Ni interferes with root maturation by disrupting the hormonal balance (Barcelo and Poschenrieder 1990). Similar observations were reported by Al-Khatib et al. (2008) after Pb treatment of tobacco roots. The presence of abnormal cortical and stele cells by Ni treatment is in accordance with findings of Kuno (1984) in mulberry roots. In *B. napus*, we see a dramatic effect in the central cylinder of Con-II roots: a significant increase in thickness by Ni treatment and a gradient approximation to control roots from CA > EDTA > CA + EDTA. It has to be taken into account that we do not distinguish between xylem and phloem cells nor cell number of the respective tissues. Other metals than Ni, on the other hand, cause a narrowing of xylem vessels, as reported for cadmium stress in *Pisum sativum* (Wong et al. 1988).

Semi-quantitative amounts of Ni at three defined distance points from the tip (50, 150, and 300 μm) as well as line scans covering the first 600 μm of root tips reflect the distribution of Ni in investigated *B. napus* varieties. The data gathered from the X-ray microanalysis points towards an increased mobility of Ni in the sensitive *B. napus* cultivar Oscar. The general level of the count curves is lower in those samples after Ni treatment, especially closer to the tip. Our results indicate that the chelating agents CA and EDTA are promoting and accumulating Ni as well as influencing their translocation. Here, the positive role of EDTA in this respect was stronger than of CA, meaning that chelated heavy metals with EDTA are mobile (Huang et al. 1997). This Ni mobility via chelation with EDTA and CA in the root tip of *B. napus* has been reported before (Epstein et al. 1999; Vassil et al. 1998). Also, in other species like *Helianthus annuus* or *Brassica juncea*, EDTA significantly increased the translocation of metals within the plants (Hsiao et al. 2007; Turgut et al. 2004). In general, the extraction of metals from the soil into the plant in phytoremediation is also greatly improved by the addition of chelating agents (Freitas et al. 2014), taking also advantage of the fact that chelators have a high affinity to many different metals (Nowack and VanBriesen 2005). Therefore, chelators minimize the amount of free Ni ions (Table 1) but this is not relevant for metal uptake and translocation within the plant. By contrast, as shown by ICP-OES, chelating agents enhance total Ni amounts in canola roots (Fig. 3). In the present study, free Ni ions were available from the time of sowing until the application of chelating agents 2 weeks later. Our treatments therefore reflect both, the effect of free Ni ions and enhanced uptake of complexed Ni by chelating agents. However, at the cellular level, the effect of free Ni ions versus chelated Ni remains to be elucidated.

As polarized molecules and metal ions cannot easily pass the plasma membrane, they are retained at the cell wall. Therefore, the cell wall is the first barrier to protect a plant from metal toxicity. An expanded cell wall area therefore favors high metal retention and metal allocation in the cell wall as an important heavy metal-tolerant mechanism (Seregin and Kozhevnikova 2006; Wojcik et al. 2005). Hence, the capacity of the cell wall for binding metals allows the plant to tolerate heavy metals. In our study, this is reflected by the highly significant increase in root radius of the Ni-tolerant *B. napus* variety Con-II.

## Conclusions

Ni treatment led to a significant increase in the total Ni amount, and in the root radius and the central cylinder, as well as the thickness of the root cortex in the Ni-tolerant variety Con-II of canola. Hence, this increased thickness of roots can be linked to Ni tolerance. Amelioration of Ni stress by the chelating agents CA and EDTA showed that only a combined treatment of CA + EDTA led to an increase in root radius in the Ni-sensitive variety Oscar. CA or EDTA by its own is not effective. *B. napus* is a widely used crop plant with metal tolerance and phytoremediation properties. Here, we see that the latter are reflected by root diameter and can be partially influenced by chelating agents.
